# Development and Validation of a Prognostic Gene-Expression Signature for Lung Adenocarcinoma

**DOI:** 10.1371/journal.pone.0044225

**Published:** 2012-09-07

**Authors:** Yun-Yong Park, Eun Sung Park, Sang Bae Kim, Sang Cheol Kim, Bo Hwa Sohn, In-Sun Chu, Woojin Jeong, Gordon B. Mills, Lauren Averett Byers, Ju-Seog Lee

**Affiliations:** 1 Department of Systems Biology, The University of Texas MD Anderson Cancer Center, Houston, Texas, United States of America; 2 Institute for Medical Convergence, Yonsei University College of Medicine, Seoul, Korea; 3 The University of Texas Graduate School of Biomedical Sciences, Houston, Texas, United States of America; 4 Korean Bioinformation Center, Korea Research Institute of Bioscience and Biotechnology, Daejeon, Korea; 5 Department of Life Science, Division of Life and Pharmaceutical Sciences, Center for Cell Signaling and Drug Discovery Research, Ewha Womans University, Seoul, Korea; 6 Division of Cancer Medicine, Department of Thoracic/Head and Neck Medical Oncology, The University of Texas MD Anderson Cancer Center, Houston, Texas, United States of America; University of Porto, Portugal

## Abstract

Although several prognostic signatures have been developed in lung cancer, their application in clinical practice has been limited because they have not been validated in multiple independent data sets. Moreover, the lack of common genes between the signatures makes it difficult to know what biological process may be reflected or measured by the signature. By using classical data exploration approach with gene expression data from patients with lung adenocarcinoma (n = 186), we uncovered two distinct subgroups of lung adenocarcinoma and identified prognostic 193-gene gene expression signature associated with two subgroups. The signature was validated in 4 independent lung adenocarcinoma cohorts, including 556 patients. In multivariate analysis, the signature was an independent predictor of overall survival (hazard ratio, 2.4; 95% confidence interval, 1.2 to 4.8; *p* = 0.01). An integrated analysis of the signature revealed that *E2F1* plays key roles in regulating genes in the signature. Subset analysis demonstrated that the gene signature could identify high-risk patients in early stage (stage I disease), and patients who would have benefit of adjuvant chemotherapy. Thus, our study provided evidence for molecular basis of clinically relevant two distinct two subtypes of lung adenocarcinoma.

## Introduction

Lung cancer is one of the most common cancers worldwide, accounting for an estimated 226,160 new cases and 160,340 deaths in 2012 in the United States alone [1]. The vast majority of lung cancers are non-small cell lung cancers (NSCLCs), of which adenocarcinoma is the most common histology (approximately 50% of all NSCLCs) [2].

The American Joint Committee on Cancer (AJCC) staging system is currently used to guide treatment decisions and is the best predictor of prognosis for patients with NSCLC. Although surgical resection is potentially curative and the most effective treatment for patients with early-stage NSCLC, 35% to 50% of patients with AJCC-defined stage I disease will experience a recurrence within 5 years [3]–[5]. This indicates that NSCLC is a very heterogeneous cancer even in the earliest stage, and this underlying heterogeneity is not well-reflected in the current staging system. Small fraction of NSCLC patients have an underlying EGFR mutations or EML4-ALK fusion which are associated with relatively high response rates to targeted molecular therapies [6]–[8]. However, for the majority of adenocarcinoma patients, we do not yet have any validated biomarkers to predict overall outcome or to guide treatment selection. Thus, to improve patient care and management, it is important to further characterize molecular subgroups significantly associated with this differential response to standard treatment and to develop models to predict those who would receive greatest benefit from these treatments.

Recent advances in technology allow unbiased genome-wide screening of potential markers or gene-expression signatures that might reflect prognosis. This approach has shown potential success in identifying prognostic and predictive markers in breast cancer [9]. Similar approaches have been applied to NSCLC and prognostic or predictive molecular signatures that may be clinically useful have been found [10]–[29]. However, the majority of these studies are limited by a lack of validation with large and multiple independent cohorts, or lack of a statistical test for the robustness of the predictive models and their contribution as new markers in prediction improvement [30]. In the current study, we applied a genome-wide survey of gene-expression data to distinguish subgroups of lung adenocarcinoma with distinct biological characteristics associated with prognosis and then identify a gene-expression signature that best reflects the biological and clinical characteristics of each subgroup. We further tested the robustness of our new prognostic gene-expression signature using several statistical approaches and multiple independent cohorts. Finally, we performed pathway analysis to study the biological differences that characterize each group.

## Methods

### Patients and Gene Expression Data

All clinical and gene expression data were collected previously and are available from public databases. Gene expression and clinical data from the National Cancer Institute (NCI) Director’s Challenge Consortium were obtained from the caArray database at the NCI (https://caarraydb.nci.nih.gov/caarray; experiment ID, jacob-00182). This data set consisted of 4 different patient cohorts, including Toronto/Canada (TC, n = 82), Memorial Sloan-Kettering Cancer Center (MSKCC, n = 104), H. Lee Moffit Cancer Center (HLM, n = 79), and University of Michigan Cancer Center (UM, n = 177) [18]. For exploration and the discovery of a potential prognostic gene-expression signature and validation of the signature, patients were divided into 2 groups. Patients from the TC and MSKCC cohorts were combined for discovery of the signature (TM cohort, n = 186). Patients from the HLM and UM cohorts were used as the first validation set (HM cohort, n = 256). Gene-expression and clinical data from Massachusetts General Hospital (MGH cohort, n = 125) were obtained from the public website of the Broad Institute (http://www.broadinstitute.org/mpr/lung) [11] and used as a second validation set. The data from the Duke Institute for Genome Sciences and Policy (Duke cohort, n = 58) were obtained from the public website of Duke University (http://data.cgt.duke.edu/oncogene.php) [22] and used as a third validation set. The data from Aichi Cancer Center (ACC cohort, n = 117) were obtained from the National Center for Biotechnology Information (NCBI) Gene Expression Omnibus (GEO) database (http://www.ncbi.nlm.nih.gov/geo, accession number GSE13213) [21] and used as the fourth validation set.

Although overall survival (OS) and recurrence free survival (RFS) were available for the NCI Director’s Challenge cohorts (TM and HM), only OS data were available for remaining cohorts (MGH, Duke, and ACC). Adjuvant chemotherapy data were available only for the TM, HM, and ACC cohorts. Of the 442 patients in TM and HM cohorts, 89 (39 in AJCC stage I, 27 in stage II, 22 in stage III, and 1 with unknown stages) received standard adjuvant chemotherapy. The remaining patients did not receive chemotherapy (n = 233) or treatment data were not available (n = 120). No patient in the ACC cohort received adjuvant chemotherapy. RFS was defined in a previous study as the time from surgery to the first confirmed relapse and was censored when a patient died or was alive without recurrence at last contact. **Table 1** shows the pathological and clinical characteristics of the patients in all 5 cohorts. All patients had undergone surgical resection as their primary treatment.

**Table 1:** Clinical and Pathological Features of Lung Adenocarcinoma Cancer Patients.

Variable		TMCohort	HMCohort	MGHCohort	DukeCohort	ACCCohort
		(Exploration cohort)	(Validation cohort 1)	(Validation cohort 2)	(Validation cohort 3)	(Validation cohort 4)
**Number of patients**		186	256	125	58	117
	Men	83 (44.6%)	140 (54.7%)	53 (42.4%)	27 (46.6%)	60 (51.3%)
	Women	103 (55.4%)	116 (45.3%)	72 (57.6%)	31 (53.4%)	57 (48.7%)
						
**Age (years)**	Median	64	66	64	67	61
	Range	35–82	33–87	33–88	43–83	31–84
						
**Disease stage**	I	119 (64.0%)	158 (61.7%)	76 (60.8%)	34 (58.6%)	79 (67.5%)
	II	46 (24.7%)	49 (19.2%)	24 (19.2%)	7 (12.1%)	13 (11.1%)
	III	21 (11.3%)	47 (18.4%)	10 (8.0%)	14 (24.1%)	25 (21.4%)
	IV	0 (0%)	0 (0%)	15 (12.0%)	3 (5.2%)	0 (0%)
	NA		2 (0.7%)			
**Adjuvant chemotherapy**						
	Yes	56 (30.1%)	33 (12.9%)	0	0	0
	No	96 (51.6%)	137 (53.5%)	0	0	117 (100%)
	NA	34 (18.3%)	86 (33.6%)	125 (100%)	58 (100%)	0
						
**Number of deaths**		74	162	71	32	49

Abbreviations: TM, Toronto and Memorial Sloan-Kettering Cancer Center; HM, H. Lee Moffit Cancer Center and University of Michigan; MGH, Massachusetts General Hospital; ACC, Aichi Cancer Center; NA, Not available.

### Statistical Analysis of Microarray Data

Biometric Research Branch (BRB)-ArrayTools were used for statistical analysis of the gene-expression data [31], and all other statistical analyses were performed in the R language environment (http://www.r-project.org). Except for data from the ACC cohort, all gene-expression data were generated using the Affymetrix (Santa Clara, CA) platform (U95A for the MGH cohort, U133A for the TM and HM cohorts, and U133 plus 2.0 for the Duke cohorts). Raw data from the Affymetrix platform were downloaded from public databases and normalized using a robust multi-array averaging method [32]. Data from the ACC cohort were generated using the Agilent whole-genome microarray platform, and pre-normalized data were downloaded and used for analysis.

We identified genes that were differentially expressed between the 2 classes using a random-variance t-test. Differences in gene expression between the 2 classes were considered statistically significant if their *p* value was less than 0.001. Cluster analysis was performed with Cluster and Treeview [33]. To predict the class of the independent patient cohort, we adopted a previously developed model [34]–[36]. Briefly, gene-expression data in the training set (the TM cohort) were combined to form a series of classifiers according to the compound covariate predictor (CCP) algorithm as described in previous publications [37] and the robustness of the classifier was estimated by the misclassification rate determined during leave-one-out cross-validation (LOOCV) of the training set. When applied to the independent validation sets, prognostic significance was estimated by evaluating the differences between Kaplan-Meier plots and log-rank tests between the 2 predicted subgroups of patients. After LOOCV, the sensitivity and specificity of the prediction models were estimated by the fraction of samples correctly predicted.

Multivariate Cox proportional hazard regression analysis was used to evaluate independent prognostic factors associated with survival, and we used gene signature, tumor stage, and pathologic characteristics as covariates. For each clinical variable, Harrell's concordance index (*c*-index) was calculated as a measure of predictive accuracy [38]. Interpretation of the *c*-index is similar to that of the area under a receiver operating characteristic curve. The higher the *c*-index, the more informative the variable is about a patient's outcome. The *c*-index analysis was carried out using the Harrell Miscellaneous (HMISC) package in the R language environment. The confidence interval (CI) of the *c*-index was estimated using 1000 bootstrap resamplings. A *p* value of less than 0.05 was considered statistically significant, and all tests were 2-tailed.

### Gene Network Analysis

IngenuityTM Pathways Analysis (IPA, Ingenuity Systems®) was used for gene network analysis. Gene network analysis was carried out by using a global molecular network developed from information contained in the Ingenuity knowledge Base. Out of 470 gene features, 468 were mapped to the Ingenuity Knowledge Base. Identified gene networks were ranked according to scores provided by IPA. The score is the likelihood of a set of genes being found in the networks due to random chance. For example, a score of 3 indicates that there is a 1/1000 chance that the focus genes are in a network due to random chance.

## Results

### Discovery, Development, and Validation of a Prognostic Gene Expression Signature

**Figure 1:**
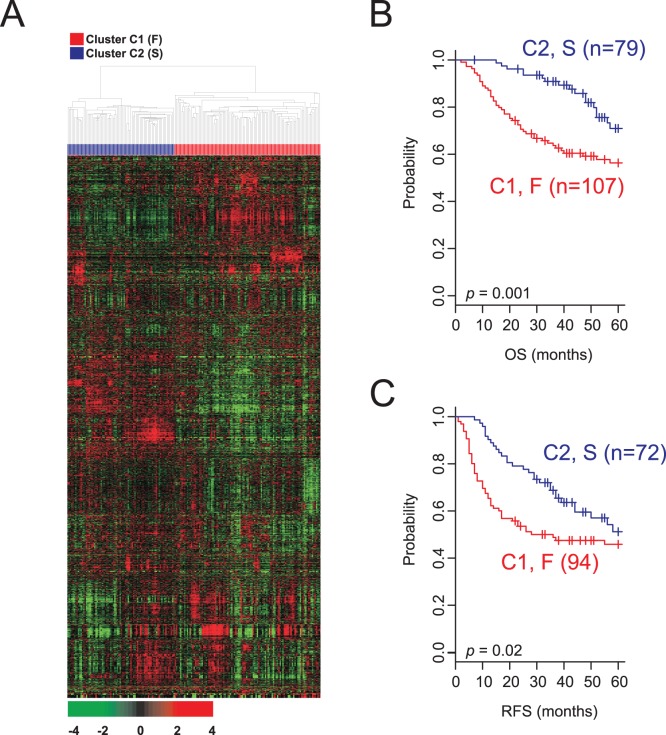
Hierarchical clustering analysis of gene expression data from the discovery cohort. (A) Hierarchical clustering of gene-expression data from 186 patients with lung adenocarcinoma in the discovery (Toronto/Canada and Memorial Sloan-Kettering Cancer Center [TM]) cohort. Genes with an expression level that was at least 2-fold different from the median value across tissues in at least 20 tissues were selected for hierarchical clustering analysis (3036 gene features). The data are presented in matrix format, where each row represents an individual gene and each column represents a tissue. Each cell in the matrix represents the expression level of a gene feature in an individual tissue. The red and green color in the cells reflects the genes’ relatively high and low expression levels, respectively, as indicated in the scale bar (a log2-transformed scale). Kaplan-Meier plots of the (B) overall survival (OS) and (C) recurrence-free survival (RFS) of patients with lung adenocarcinoma in the TM cohort. Patients were stratified according to gene-expression patterns (creating two clusters, C1 and C2). RFS data are currently not available from 20 patients.

To find potential prognostic subgroups of lung adenocarcinoma with distinct biological characteristics, we collected gene expression data from previous studies and divide them into 5 independent cohorts (one exploration cohort and 4 validation cohorts) (**Table 1**). Hierarchical clustering analysis of the gene expression data from the exploration data set (TM cohort, n = 186) revealed 2 distinct subgroups (clusters) of lung adenocarcinoma (**Fig. 1A**). Subsequent analysis of the clinical data showed a significant difference in clinical outcomes between the 2 subgroups. The OS rates of patients in cluster C1 were significantly lower than those of patients in cluster C2 (3-year survival rate: 63.7% [cluster C1] vs 90.1% [cluster C2]; *p* = 1.5×10^−5^ by χ^2^-test). The hazard ratio (HR) for death of cluster C1 was 2.36 (95% CI, 1.35 to 4.13; *p* = 0.002). The significance trend remained the same for RFS (3-year RFS rate: 48.8% [cluster C1] vs 68.7% [cluster C2]; *p* = 0.009 by χ^2^-test). The HR for recurrence of cluster C1 was 1.58 (95% CI, 1.01 to 2.46; *p* = 0.04). Continuous survival analysis verified that the patients in cluster C2 had significantly better OS and RFS than those in cluster C1 (*p* = 0.001 for OS and *p* = 0.02 for RFS, by log-rank test; **Fig. 1B and 1C**).

We next sought to identify a limited number of genes whose expression was tightly associated with the 2 subgroups. By applying a stringent threshold cutoff (*p*<0.001 and at least a 2-fold difference between subgroups), we identified 193 gene features differentially expressed between 2 subgroups (**Fig. S1 and Table S1**). Of note, the expression of many genes involved in cell proliferation and cell cycle regulation, such as *CCNB1*, *TOP2A*, *AURKA*, *CDC2*, and *FOXM1,* was significantly higher (p<0.001, by t-test) in patients in the poor-prognosis subgroup (C1), indicating that tumors in the C1 subgroup had higher cell proliferation rates. Thus, we renamed the 2 clusters C1 and C2 as cluster F (for “fast-growing tumors”) and cluster S (for “slow-growing tumors”), respectively.

### Independent Validation of the Identified Expression Signature

**Figure 2:**
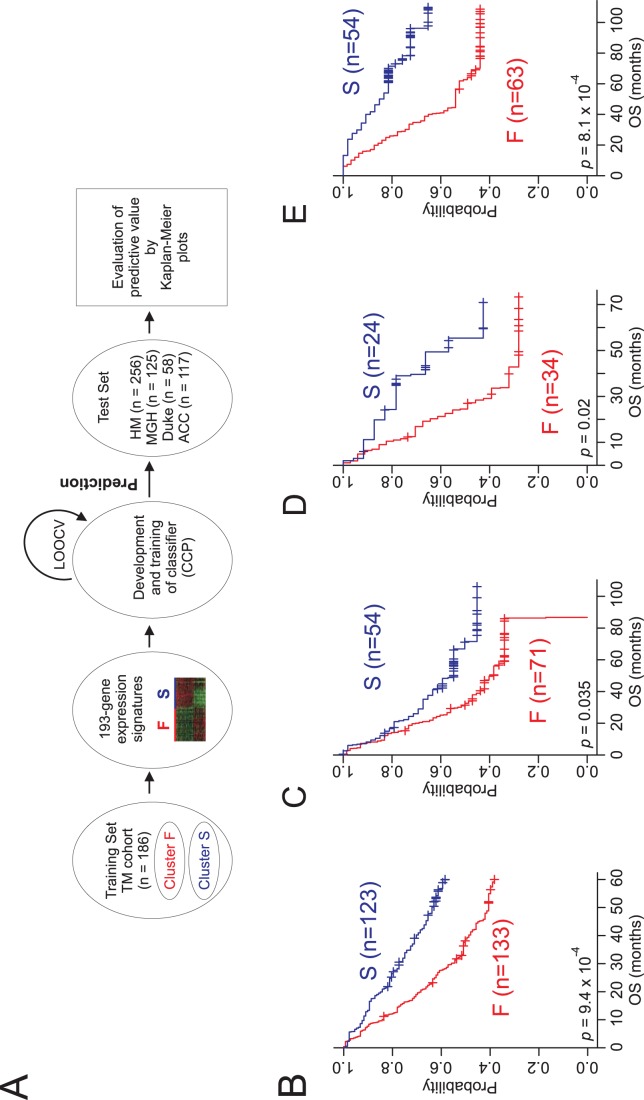
Construction of the prediction model and evaluation of predicted outcome. (A) Schematic overview of the strategy used for constructing prediction models and evaluating the predicted outcomes based on gene expression signatures. Kaplan-Meier plots of the overall survival (OS) of the 2predicted groups of lung adenocarcinoma patients in the (B) HM, (C) MGH, (D) Duke, (E) and ACC cohorts. The differences between groups were significant, as indicated by the log-rank test. The + symbols in panels B–E indicate censored data.

With a gene expression signature (193 genes) that accurately reflected prognosis in TM cohort, we next sought to validate the association of the gene signature with prognosis in 4 independent patient cohorts (HM, MGM, Duke, and ACC cohort). For this validation, previously established data training and prediction methods [34]–[36] were applied to gene expression data from the HM cohort (n = 256; **Fig. 2A**). When lung adenocarcinoma patients in the HM cohort were stratified according to the prognostic gene expression signature, Kaplan-Meier plots showed significant differences in OS (*p* = 9.4×10^−4^ by log-rank test) between the 2 subgroups of patients that were predicted by the CCP (**Fig. 2B**). The specificity and sensitivity for correctly predicting subgroup F during LOOCV were 0.881 and 0.975, respectively.

To assess the robustness of our gene-expression signature, we applied our prediction method to 2 additional independent validation cohorts (MGH cohort, n = 125; Duke cohort, n = 58). Consistent with the results from the HM cohort, the expression signature successfully discriminated patients with poor prognosis (subgroup F) from those with a better prognosis (subgroup S; **Fig. 2C and 2D**). In addition, we further tested the robustness of the signature using another independent cohort with a different ethnic background, that is, the 117 Japanese patients with lung adenocarcinoma from the ACC cohort [21]. When patients in the ACC cohort were stratified according to their gene expression signatures, Kaplan-Meier plots showed significant differences in OS (*p* = 8.1×10^−4^ by log-rank test) between the 2 predicted subgroups (**Fig. 2E**). Taken together, these results demonstrated the robustness of the gene signature for identifying patients at high risk for disease recurrence and poorer survival.

### Significant Association of the Gene Signature with Clinical Variables

To evaluate the prognostic value of the gene expression signature in combination with other clinical variables, including patient age at diagnosis, disease stage by AJCC criteria, smoking status, sex, and mutation status of certain oncogenes and tumor suppressor genes (i.e., *KRAS*, *EGFR*, and *TP53*), univariate and multivariate Cox proportional hazards regression analyses were performed in the ACC cohort. All patients in this cohort received uniform treatment (curative resection without adjuvant chemotherapy) thus minimizing confounding factors associated with different treatments. In the univariate analysis, both disease stage and the gene-expression signature were significantly associated with OS (*p* = 2.17×10^−4^ and *p* = 0.001, respectively). In the multivariate analysis, disease stage and gene expression signature maintained their significance (*p* = 0.002 and *p* = 0.01, respectively; **Table 2**).

**Table 2:** Univariate and Multivariate Cox Proportional Hazard Regression Analyses of Overall Survival in the ACC Cohort (n = 117).

	Univariate		Multivariate	
Variable	Hazard Ratio (95% CI)	*p*	Hazard Ratio (95% CI)	*P*
**Sex** **(M vs F)**	1.36 (0.77–2.38)	0.28	1.52 (0.66–3.4)	0.31
**Age**	1.0 (0.97–1.03)	0.67	1.0 (0.97–1.03)	0.68
**EGFR** **(mutant ** ***vs*** ** WT)**	1.0 (0.57–1.8)	0.95	1.5 (0.77–2.8)	0.23
**KRAS** **(mutant vs WT)**	1.5 (0.7–3.2)	0.27	1.3 (0.57–3.3)	0.5
**TP53** **(mutant vs WT)**	1.35 (0.76–2.4)	0.29	1.1(0.6–2.0)	0.72
**Smoking** **(yes vs no)**	1.36 (0.77–2.4)	0.28	0.76 (0.33–1.7)	0.53
**Disease stage** **(I, II, III)**	1.78 (1.3–2.4)	2.17×10^−4^	1.65 (1.2–2.2)	0.002
**Gene signature** **(F vs S)**	2.76 (1.4–5.1)	0.001	2.4 (1.2–4.8)	0.01

Abbreviations: CI, confidence interval; M, male; F (sex), female; WT, wild-type; F (gene signature), fast-growing; S, slow-growing.

In addition to performing multivariate analysis, we assessed our new prognostic signature’s potential using the “drop in concordance index” approach [30], [39]. Briefly, we generated prediction models using all clinical variables used in the multivariate analysis. While the best model was constructed using all of the variables, test models each lacking 1 variable were generated and compared with the best model. In each comparison, the predictive value of each variable was weighted by measuring the decreased value of the *c*-index in each test model. Omission of the gene signature in the prediction model caused the largest decrease in the *c*-index value (**Table S2**), suggesting that the signature not only retains its prognostic relevance over the classical pathological prognostic features but also significantly improves the prediction accuracy.

The independence of the new prognostic gene expression signature over the current staging system was further supported by analysis of pooled data from all 4 validation cohorts (n = 556). As expected, the OS of subgroup F was significantly worse than that of subgroup S (*p* = 3.0×10^−8^ by log-rank test) when all patients were included in the analysis (**Fig. S2B**). In subset analysis, the gene-expression signature successfully identified poorer survival for both stage I (*p* = 0.006 by log-rank test) and stage II patients (*p* = 0.03 by log-rank test; **Fig. S2C and S2D**). Taken together, these findings strongly demonstrate that our new prognostic gene-expression signature is independent from the current staging system.

### Association of the Gene Signature with Potential Benefit from Adjuvant Chemotherapy

**Figure 3:**
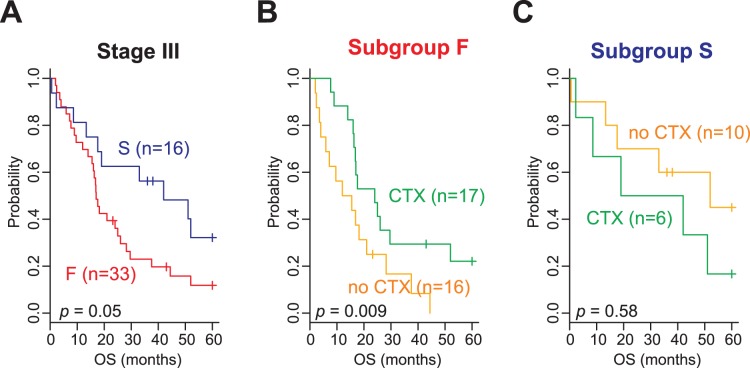
Significant association of the 2 gene-expression signature subtypes with adjuvant chemotherapy. (A) Kaplan-Meier plots of the overall survival (OS) of adenocarcinoma patients in the TM and HM cohorts. The data were plotted according to the prognostic gene-expression signature (subgroups F and S). Kaplan-Meier plots of patients in (B) subgroup F or (C) subgroup S with stage III disease. Data were plotted according to whether patients were treated with or without adjuvant chemotherapy (CTX).

Of the 442 patients from TM and HM cohorts, adjuvant chemotherapy data were available for 322 patients. Thus, we next sought to determine whether the new gene expression signature could predict a potential benefit from adjuvant chemotherapy. To examine the association of the gene signature with response to adjuvant chemotherapy, we performed subset analysis with patients in AJCC stage III, a stage for which the benefit of adjuvant chemotherapy has been previously demonstrated [40]–[42]. Patients with stage III disease (n = 49) were subdivided into 2 subgroups (F or S), and the difference in OS was independently assessed. Adjuvant chemotherapy significantly affected OS in patients in subgroup F (3-year OS rate, 29.4% [adjuvant chemotherapy] vs 16.7% [no adjuvant chemotherapy]; *p* = 0.009 by log-rank test; **Fig. 3B**). However, there was not a significant benefit from adjuvant chemotherapy for patients in subgroup S (3-year OS rate, 50% [adjuvant chemotherapy] vs 60% [no adjuvant chemotherapy]; *p* = 0.58 by log-rank test; **Fig. 3C**). When a Cox regression model was applied, the interaction of subgroups with adjuvant chemotherapy reached a significance level of 0.03. Consistent with the Kaplan-Meier plot and log-rank test, the estimated HR for death for adjuvant chemotherapy in subgroup F was 0.44 (95% CI, 0.2 to 0.95; *p* = 0.036), while the HR for death for adjuvant chemotherapy in subgroup S was 1.96 (95% CI, 0.56 to 6.88; *p* = 0.29). This suggests a benefit of adjuvant therapy only in the F subgroup and potential harm associated with adjuvant treatment in the S subgroup. A similar trend was observed in the Stage II patients, although it did not reach statistical significance (*p* = 0.22) (**Fig. S3**). In the Stage I patients, there was an overall trend towards worse outcome with adjuvant chemotherapy (**Fig. S3**).

### Biological Insights from the Conserved Prognostic Gene-Expression Signature

**Figure 4:**
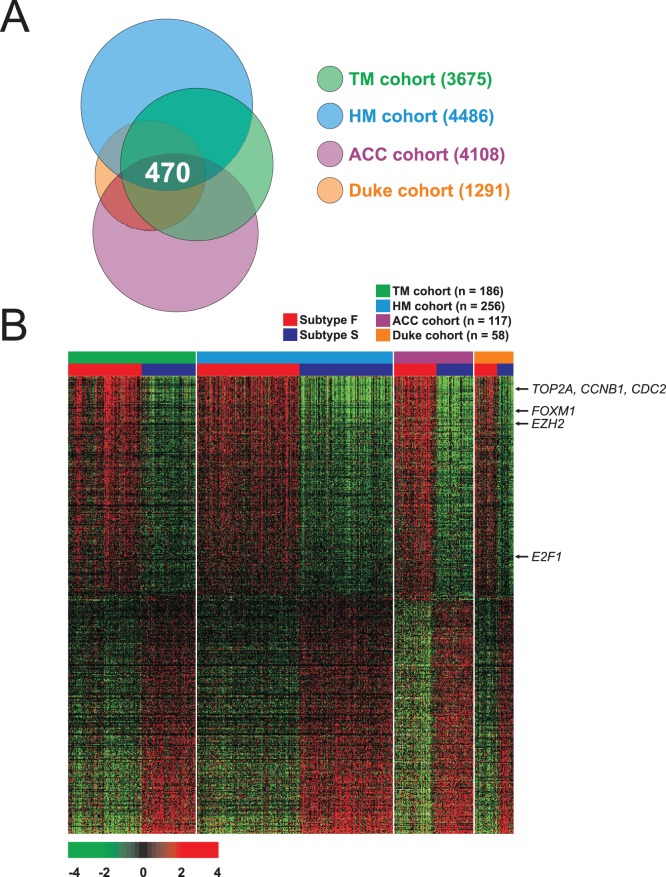
Cross comparison of gene lists from 4 independent cohorts of lung adenocarcinoma patients. (A) Venn diagram of genes whose expression is significantly different between subgroups F and S. a univariate test (2-sample t-test) with multivariate permutation test (10,000 random permutations) was applied. In each comparison, we applied a cut-off P-value of less than 0.001 to retain genes whose expression was significantly different between the 2 groups of tissues examined. (B) Expression patterns of selected genes shared in 4 lung adenocarcinoma cohorts. The expression of 470 genes is commonly up- or down-regulated in all 4 cohorts. Colored bars at the top of the heat map represent samples as indicated.

To elucidate the biological characteristics of the subgroup with poor prognosis (subgroup F), we attempted to identify genes whose expression differed between the “F” and “S” subgroups across all data sets. We excluded gene-expression data from the MGH cohort in this analysis to maximize the compatibility of the data sets, since the MGH data were generated using an old microarray platform (U95A) with a limited number of gene probes. We applied a stringent cut-off (*p*<0.001) to avoid inclusion of potential false-positive genes. When they were all compared together, 470 genes were shared by all 4 cohorts (**Fig. 4A**).

We next performed pathway analysis on the 470 genes using the Ingenuity Pathway Analysis tool that is a controlled vocabulary-based pathway tool. This analysis revealed a series of putative networks. Functional connectivity of the top network revealed a strong over-representation of the *E2F1* pathway in patients in the F subgroup (**Fig. S4**), suggesting that its activation may be a key genetic determinant associated with the poorer survival of lung adenocarcinoma patients in this subgroup. Expression of *EZH2*, which is frequently overexpressed in many cancers [43], was also significantly higher in subgroup F, indicating the importance of the *E2F1*-*EZH2* network in the progression of lung adenocarcinoma. *TP53* was overrepresented in another network (**Fig. S5**). Interestingly, many genes negatively regulated by *TP53* were overexpressed in the *TP53* networks. For example, previous studies have demonstrated that expression of *PRC1* and *BUB1* are directly suppressed by *TP53* [44], [45], but their expression is significantly upregulated in subgroup F, suggesting that the biological activity of *TP53* may be substantially lost in this subgroup.

## Discussion

By analyzing gene-expression data from lung adenocarcinoma tissues, we identified a limited number of genes (193 genes) whose expression is significantly associated with prognosis. The robustness of this gene-expression signature was validated in 4 independent cohorts with a total of 556 patients. Since current staging systems and biomarkers are limited in their ability to assess risk of recurrence and benefit from adjuvant chemotherapy in lung adenocarcinoma, our new gene-expression signature may represent a tool that could help further refine treatment decisions based on the tumors’ molecular profiles.

For development and validation of a robust, prognostic gene expression signature, we applied 2 independent but complementary methods. Unsupervised hierarchical clustering was first applied to identify subgroups with significant differences in biological characteristics as well as prognosis. In the second approach, supervised prediction models were applied to validate the association of the signature with clinical outcomes in 4 independent patient cohorts. The robustness of the 193-gene signature was supported by the high sensitivity (>0.9) and specificity (>0.8) values seen during training of the prediction models within the discovery cohort and a significant association between the predicted outcome and patient prognosis in 4 test cohorts. In addition to its robustness, the prognostic gene signature’s independence as a prognostic marker was supported by the results of vigorous tests using various approaches. First, the signature could identify high-risk patients among those with early stage adenocarcinoma (stage I and II). Second, in multivariate analysis, the signature was one of the most significant predictive factors for OS. Third, the signature was the most significant contributor to the predicted OS in models using the drop-in *c*-index approach. Taken together, these results strongly support that the 2 subgroups of lung adenocarcinoma predicted here are novel prognostic clinical subgroups that are not recognized by the current staging system.

Subset analysis of patients with available chemotherapy data strongly suggested that the 193-gene signature can predict which patients will benefit from adjuvant chemotherapy. In patients with stage III disease, adjuvant chemotherapy was significantly associated with improved outcome for patients in subgroup F (HR, 0.44; 95% CI, 0.2 to 0.95; *p* = 0.036), whereas its benefit was not statistically significant for patients in subgroup S (HR, 1.96; 95% CI, 0.56 to 6.88; *p* = 0.29). Thus, our newly identified gene signature showed both a prognostic and predictive association.

Interestingly, our prognostic gene expression signature lacks overlapped genes with previously identified prognostic gene expression signatures. For example, of 193 genes, only one gene is common with the prognostic signature discovered in Japanese patients [21]. Likewise, no or only few genes were shared with other signatures such as EGFR-mutation signature [29], stage I specific prognostic signature [27], and ALK-associated gene expression signature [28]. Moreover, when different signatures were compared all together in multiple-comparison manner, only few genes were shared among the signatures. Our finding is consistent with previous study in breast cancer showing absence of gene overlap although concordance of predicted outcome is very high [46].

Overexpression of *EZH2*, a methyltransferase that catalyzes H3 trimethylation on lysine 27 and is essential for stem cell self-renewal [47], in subgroup F is in good agreement with previous studies. Its altered expression has been linked to the aggressive progression of many cancers through its activation of angiogenesis and maintenance of the tumor-initiating cell (or cancer stem cell) population [48]. *EZH2* is a newly identified downstream target of *E2F1* [49], which is a major downstream effector of the *RB* tumor suppressor and has a pivotal role in controlling cell cycle progression [50]. Expression of *E2F1*’s well-known downstream target genes was significantly upregulated in subgroup F (**Fig. S4**), indicating that *E2F1* was highly activated in subgroup F and that *E2F1*-mediated regulation of *EZH2* may be a key genetic event associated with poor prognosis in lung adenocarcinoma.

Expression of *TYMS* (thymidylate synthase) was also higher in subgroup F, which is in good agreement with previous studies showing that higher expression of *TYMS* is significantly associated with poorer prognosis in lung adenocarcinoma [51], [52]. Pemetrexed, a potent inhibitor of TYMS [53], has emerged as one of the most active agents for the treatment of patients with advanced NSCLC. Previous studies have demonstrated that higher *TYMS* expression is associated with a lower chemotherapeutic effect of pemetrexed in patients with a variety of solid tumors [54]–[56] and forced overexpression of *TYMS* in NSCLC cells reduced sensitivity to pemetrexed [57]. Since expression of *TYMS* is significantly higher in subgroup F, our data suggest that pemetrexed may show limited antitumor activity for patients in this subgroup. By contrast, patients in subgroup S may benefit from pemetrexed because they have lower expression of *TYMS*. Thus, the 2 newly identified subgroups of lung adenocarcinoma not only well reflect previously recognized clinical characteristics of lung adenocarcinoma but may also provide guidance for treatment regimens.

In a recent evaluation of all prognostic gene expression signatures for lung cancer [39], [58], 2 important criteria were suggested for a new prognostic signature to be accepted by the medical community. First, the new signature should be rigorously tested for statistical validation and reproducibility in large multiple-patient cohorts. Second, the new signature should show good predictive power over and above current risk factors. Our prognostic signature fulfills these 2 suggested criteria, as evidenced by validation of the signature in 4 independent cohorts (a total of 556 patients), independence from the current staging system, improvement of predictive power when included in the prediction model, and identification of high risk-patients with very early-stage disease. Although interesting, our analysis has some limitations because we only used mRNA expression level of genes that is not always correlated with their biological activity. Thus, other approaches better reflecting biological activity like proteomics should be used for finding better functional markers in future study.

In conclusion, using gene-expression data from multiple cohorts, we identified 2 new prognostic subgroups of lung adenocarcinoma that show significant differences in patient survival. The 193-gene signature can identify patients with a high risk of recurrence, as well as patients who would have benefited from adjuvant chemotherapy. This study clearly demonstrated that our gene-expression signature reflects the molecular characteristics of different subgroups of lung adenocarcinoma and provides an opportunity to rationally design future clinical trials so that patients who might benefit from adjuvant chemotherapy can be identified. Our results, if confirmed in prospective studies, may improve patient care by providing more practical guidance for treatment.

## Supporting Information

Figure S1 **Genes differentially expressed between cluster C1 (F) and cluster C2 (S) in TM cohort (n = 186).** Genes were selected by univariate test (2-sample t-test) with multivariate permutation test and stringent cut-off (P<0.001 and >2-fold difference) was applied to retain genes whose expression is significantly different between the 2 groups of tissues examined (193 genes). The data are presented in matrix format, where rows represent individual gene and columns represent each tissue. Each cell in the matrix represents the expression level of a gene feature in an individual tissue. The red and green color in cells reflect relative high and low expression levels respectively as indicated in the scale bar (log2 transformed scale).
(EPS)

Figure S2 **Kaplan-Meier plots of the overall survival (OS) in patients in all validation cohorts.** Patients were stratified by (A) disease stage or (B) gene expression signature. Subset analysis showed that the gene expression signature was predictive in patients with (C) stage I or (D) stage II disease. Of 556 patients, stage data are not available from 2 patients.
(EPS)

Figure S3 **Kaplan-Meier plots of the overall survival (OS) in patients with Stage I and Stage II** **disease in TM and HM cohorts.** The data were plotted according to whether patients were treated with or without adjuvant chemotherapy (CTX). (A) Subtype F in stage I. (B) Subtype S in stage I. (C) Subtype F in stage II. (D) Subtype S in stage II.
(EPS)

Figure S4 **E2F1 networks in F subgroup of lung adenocarcinoma.** Ingenuity® pathway analysis revealed that networks of genes considerably associated with the E2F1in conserved gene expression data from the 4 cohorts. Upregulated and downregulated genes in the F subgroup are indicated by red and green, respectively. The lines and arrows represent functional and physical interactions and the directions of regulation from the literature.
(EPS)

Figure S5 **TP53 networks the in F subgroup of lung adenocarcinoma.** Ingenuity® pathway analysis revealed that networks of genes considerably associated with the TP53 in conserved gene expression data from the 4 cohorts. Upregulated and downregulated genes in the F subgroup are indicated by red and green, respectively. The lines and arrows represent functional and physical interactions and the directions of regulation from the literature.
(EPS)

Table S1 **Summary of 193 gene features in prognostic expression signature.**
(DOCX)

Table S2 **Drop in Concordance-index Score of Clinical Variables in ACC Cohort.**
(DOCX)
