# Supplementary material for: Development and Validation of a Prognostic Gene-Expression Signature for Lung Adenocarcinoma
Source: PLoS One. 2012 Sep 7;7(9):e44225. doi: 10.1371/journal.pone.0044225 (PMC3436895; doi:10.1371/journal.pone.0044225)
Supplement: Table S1 — Summary of 193 gene features in prognostic expression signature. (DOCX) [file pone.0044225.s006.docx]

**Table S1.** Summary of 193 gene features in prognostic expression signature

| **Affy Probe ID** | *Symbol* | UG Cluster | Entrez ID | Cyto band | F/S Ratios (Log2) |
| --- | --- | --- | --- | --- | --- |
| **37892_at** | *COL11A1* | Hs.523446 | 1301 | 1p21 | 2.11 |
| **201291_s_at** | *TOP2A* | Hs.156346 | 7153 | 17q21-q22 | 1.63 |
| **212353_at** | *SULF1* | Hs.409602 | 23213 | 8q13.2-q13.3 | 1.51 |
| **201292_at** | *TOP2A* | Hs.156346 | 7153 | 17q21-q22 | 1.48 |
| **202870_s_at** | *CDC20* | Hs.524947 | 991 | 1p34.1 | 1.47 |
| **209942_x_at** | *MAGEA3* | Hs.417816 | 4102 | Xq28 | 1.46 |
| **209773_s_at** | *RRM2* | Hs.226390 | 6241 | 2p25-p24 | 1.44 |
| **210728_s_at** | *CALCA* | Hs.37058 | 796 | 11p15.2-p15.1 | 1.43 |
| **218469_at** | *GREM1* | Hs.40098 | 26585 | 15q13-q15 | 1.43 |
| **212354_at** | *SULF1* | Hs.409602 | 23213 | 8q13.2-q13.3 | 1.41 |
| **202620_s_at** | *PLOD2* | Hs.477866 | 5352 | 3q23-q24 | 1.38 |
| **217561_at** | *CALCA* | Hs.37058 | 796 | 11p15.2-p15.1 | 1.36 |
| **201890_at** | *RRM2* | Hs.226390 | 6241 | 2p25-p24 | 1.36 |
| **202779_s_at** | *UBE2S* | Hs.396393 | 27338 | 19q13.43 | 1.34 |
| **204320_at** | *COL11A1* | Hs.523446 | 1301 | 1p21 | 1.33 |
| **214612_x_at** | *MAGEA6* | Hs.441113 | 4105 | Xq28 | 1.31 |
| **202954_at** | *UBE2C* | Hs.93002 | 11065 | 20q13.12 | 1.30 |
| **218009_s_at** | *PRC1* | Hs.567385 | 9055 | 15q26.1 | 1.29 |
| **210727_at** | *CALCA* | Hs.37058 | 796 | 11p15.2-p15.1 | 1.29 |
| **218468_s_at** | *GREM1* | Hs.40098 | 26585 | 15q13-q15 | 1.23 |
| **214710_s_at** | *CCNB1* | Hs.23960 | 891 | 5q12 | 1.21 |
| **204170_s_at** | *CKS2* | Hs.83758 | 1164 | 9q22 | 1.21 |
| **204580_at** | *MMP12* | Hs.1695 | 4321 | 11q22.3 | 1.21 |
| **212344_at** | *SULF1* | Hs.409602 | 23213 | 8q13.2-q13.3 | 1.19 |
| **202311_s_at** | *COL1A1* | Hs.172928 | 1277 | 17q21.33 | 1.18 |
| **201195_s_at** | *SLC7A5* | Hs.513797 | 8140 | 16q24.3 | 1.18 |
| **204825_at** | *MELK* | Hs.184339 | 9833 | 9p13.2 | 1.16 |
| **202619_s_at** | *PLOD2* | Hs.477866 | 5352 | 3q23-q24 | 1.15 |
| **218883_s_at** | *MLF1IP* | Hs.575032 | 79682 | 4q35.1 | 1.14 |
| **209987_s_at** | *ASCL1* | Hs.704281 | 429 | 12q23.2 | 1.14 |
| **209988_s_at** | *ASCL1* | Hs.704281 | 429 | 12q23.2 | 1.13 |
| **201037_at** | *PFKP* | Hs.26010 | 5214 | 10p15.3-p15.2 | 1.13 |
| **202095_s_at** | *BIRC5* | Hs.514527 | 332 | 17q25 | 1.13 |
| **209278_s_at** | *TFPI2* | Hs.438231 | 7980 | 7q22 | 1.13 |
| **200606_at** | *DSP* | Hs.519873 | 1832 | 6p24 | 1.12 |
| **210052_s_at** | *TPX2* | Hs.708960 | 22974 | 20q11.2 | 1.11 |
| **202998_s_at** | *LOXL2* | Hs.626637 | 4017 | 8p21.3-p21.2 | 1.10 |
| **211571_s_at** | *VCAN* | Hs.643801 | 1462 | 5q14.3 | 1.10 |
| **202589_at** | *TYMS* | Hs.592338 | 7298 | 18p11.32 | 1.09 |
| **209714_s_at** | *CDKN3* | Hs.84113 | 1033 | 14q22 | 1.09 |
| **221730_at** | *COL5A2* | Hs.445827 | 1290 | 2q14-q32 | 1.08 |
| **204026_s_at** | *ZWINT* | Hs.591363 | 11130 | 10q21-q22 | 1.08 |
| **215646_s_at** | *VCAN* | Hs.643801 | 1462 | 5q14.3 | 1.08 |
| **203819_s_at** |  |  |  |  | 1.07 |
| **218039_at** | *NUSAP1* | Hs.615092 | 51203 | 15q15.1 | 1.06 |
| **202580_x_at** | *FOXM1* | Hs.239 | 2305 | 12p13 | 1.06 |
| **202503_s_at** | *KIAA0101* | Hs.81892 | 9768 | 15q22.31 | 1.05 |
| **210559_s_at** | *CDC2* | Hs.334562 | 983 | 10q21.1 | 1.05 |
| **204033_at** | *TRIP13* | Hs.436187 | 9319 | 5p15.33 | 1.05 |
| **218542_at** | *CEP55* | Hs.14559 | 55165 | 10q23.33 | 1.04 |
| **211071_s_at** | *MLLT11* | Hs.75823 | 10962 | 1q21 | 1.04 |
| **202310_s_at** | *COL1A1* | Hs.172928 | 1277 | 17q21.33 | 1.04 |
| **204620_s_at** | *VCAN* | Hs.643801 | 1462 | 5q14.3 | 1.04 |
| **207828_s_at** | *CENPF* | Hs.497741 | 1063 | 1q32-q41 | 1.03 |
| **208079_s_at** | *AURKA* | Hs.250822 | 6790 | 20q13.2-q13.3 | 1.03 |
| **221731_x_at** | *VCAN* | Hs.643801 | 1462 | 5q14.3 | 1.02 |
| **202404_s_at** | *COL1A2* | Hs.489142 | 1278 | 7q22.1 | 1.02 |
| **203755_at** | *BUB1B* | Hs.631699 | 701 | 15q15 | 1.02 |
| **219918_s_at** | *ASPM* | Hs.121028 | 259266 | 1q31 | 1.01 |
| **201761_at** | *MTHFD2* | Hs.469030 | 10797 | 2p13.1 | 1.00 |
| **210298_x_at** | *FHL1* | Hs.435369 | 2273 | Xq26 | -1.00 |
| **206742_at** | *FIGF* | Hs.11392 | 2277 | Xp22.31 | -1.00 |
| **207761_s_at** | *METTL7A* | Hs.711113 | 25840 | 12q13.12 | -1.01 |
| **222073_at** | *COL4A3* | Hs.570065 | 1285 | 2q36-q37 | -1.01 |
| **215536_at** | *HLA-DQB2* | Hs.409934 | 3120 | 6p21 | -1.01 |
| **206311_s_at** | *PLA2G1B* | Hs.992 | 5319 | 12q23-q24.1 | -1.01 |
| **204018_x_at** |  |  |  |  | -1.02 |
| **212671_s_at** |  |  |  |  | -1.02 |
| **215783_s_at** | *ALPL* | Hs.75431 | 249 | 1p36.12 | -1.02 |
| **218627_at** | *DRAM1* | Hs.525634 | 55332 | 12q23.2 | -1.02 |
| **207980_s_at** | *CITED2* | Hs.82071 | 10370 | 6q23.3 | -1.02 |
| **204112_s_at** | *HNMT* | Hs.42151 | 3176 | 2q22.1 | -1.02 |
| **221884_at** | *MECOM* | Hs.659873 | 2122 | 3q24-q28 | -1.03 |
| **203549_s_at** | *LPL* | Hs.180878 | 4023 | 8p22 | -1.03 |
| **212195_at** | *IL6ST* | Hs.532082 | 3572 | 5q11 | -1.03 |
| **211696_x_at** | *HBB* | Hs.523443 | 3043 | 11p15.5 | -1.03 |
| **200762_at** | *DPYSL2* | Hs.173381 | 1808 | 8p22-p21 | -1.03 |
| **219869_s_at** | *SLC39A8* | Hs.288034 | 64116 | 4q22-q24 | -1.03 |
| **210982_s_at** | *HLA-DRA* | Hs.520048 | 3122 | 6p21.3 | -1.03 |
| **207542_s_at** | *AQP1* | Hs.76152 | 358 | 7p14 | -1.04 |
| **213258_at** | *TFPI* | Hs.516578 | 7035 | 2q32 | -1.04 |
| **204201_s_at** | *PTPN13* | Hs.436142 | 5783 | 4q21.3 | -1.04 |
| **216474_x_at** |  |  |  |  | -1.04 |
| **210068_s_at** | *AQP4* | Hs.315369 | 361 | 18q11.2-q12.1 | -1.04 |
| **214091_s_at** | *GPX3* | Hs.386793 | 2878 | 5q23 | -1.04 |
| **207847_s_at** | *MUC1* | Hs.89603 | 4582 | 1q21 | -1.05 |
| **207134_x_at** | *TPSB2* | Hs.405479 | 64499 | 16p13.3 | -1.05 |
| **215193_x_at** |  |  |  |  | -1.05 |
| **211654_x_at** | *HLA-DQB1* | Hs.409934 | 3119 | 6p21.3 | -1.05 |
| **209357_at** | *CITED2* | Hs.82071 | 10370 | 6q23.3 | -1.06 |
| **211699_x_at** |  |  |  |  | -1.06 |
| **209598_at** | *PNMA2* | Hs.591838 | 10687 | 8p21.2 | -1.06 |
| **221577_x_at** | *GDF15* | Hs.616962 | 9518 | 19p13.11 | -1.06 |
| **203323_at** | *CAV2* | Hs.212332 | 858 | 7q31.1 | -1.06 |
| **205683_x_at** | *TPSAB1* | Hs.405479 | 7177 | 16p13.3 | -1.06 |
| **210119_at** | *KCNJ15* | Hs.411299 | 3772 | 21q22.2 | -1.06 |
| **201348_at** | *GPX3* | Hs.386793 | 2878 | 5q23 | -1.07 |
| **200795_at** | *SPARCL1* | Hs.62886 | 8404 | 4q22.1 | -1.07 |
| **219856_at** | *C1orf116* | Hs.32417 | 79098 | 1q32.1-q32.2 | -1.07 |
| **209763_at** | *CHRDL1* | Hs.496587 | 91851 | Xq23 | -1.07 |
| **217414_x_at** |  |  |  |  | -1.07 |
| **218723_s_at** | *C13orf15* | Hs.507866 | 28984 | 13q14.11 | -1.07 |
| **204343_at** | *ABCA3* | Hs.26630 | 21 | 16p13.3 | -1.08 |
| **215300_s_at** | *FMO5* | Hs.642706 | 2330 | 1q21.1 | -1.08 |
| **209458_x_at** |  |  |  |  | -1.08 |
| **204072_s_at** | *FRY* | Hs.507669 | 10129 | 13q13.1 | -1.09 |
| **211657_at** | *CEACAM6* | Hs.466814 | 4680 | 19q13.2 | -1.09 |
| **204215_at** | *C7orf23* | Hs.719226 | 79161 | 7q21.1-q21.2 | -1.10 |
| **212097_at** | *CAV1* | Hs.74034 | 857 | 7q31.1 | -1.10 |
| **217478_s_at** | *HLA-DMA* | Hs.351279 | 3108 | 6p21.3 | -1.11 |
| **215059_at** |  |  |  |  | -1.11 |
| **203548_s_at** | *LPL* | Hs.180878 | 4023 | 8p22 | -1.12 |
| **211991_s_at** | *HLA-DPA1* | Hs.347270 | 3113 | 6p21.3 | -1.12 |
| **209373_at** | *MALL* | Hs.185055 | 7851 | 2q13 | -1.14 |
| **211745_x_at** |  |  |  |  | -1.14 |
| **211549_s_at** | *HPGD* | Hs.596913 | 3248 | 4q34-q35 | -1.15 |
| **209116_x_at** | *HBB* | Hs.523443 | 3043 | 11p15.5 | -1.16 |
| **211024_s_at** | *NKX2-1* | Hs.94367 | 7080 | 14q13 | -1.16 |
| **218100_s_at** | *IFT57* | Hs.412196 | 55081 | 3q13.12-q13.13 | -1.16 |
| **213106_at** | *ATP8A1* | Hs.435052 | 10396 | 4p14-p12 | -1.16 |
| **202291_s_at** | *MGP* | Hs.365706 | 4256 | 12p13.1-p12.3 | -1.17 |
| **205200_at** | *CLEC3B* | Hs.476092 | 7123 | 3p22-p21.3 | -1.17 |
| **205927_s_at** | *CTSE* | Hs.644082 | 1510 | 1q31 | -1.18 |
| **214414_x_at** |  |  |  |  | -1.18 |
| **210081_at** | *AGER* | Hs.534342 | 177 | 6p21.3 | -1.19 |
| **212998_x_at** | *HLA-DQB1* | Hs.409934 | 3119 | 6p21.3 | -1.20 |
| **204124_at** | *SLC34A2* | Hs.479372 | 10568 | 4p15.3-p15.1 | -1.21 |
| **212328_at** | *LIMCH1* | Hs.335163 | 22998 | 4p13 | -1.21 |
| **206385_s_at** | *ANK3* | Hs.499725 | 288 | 10q21 | -1.22 |
| **212327_at** | *LIMCH1* | Hs.335163 | 22998 | 4p13 | -1.23 |
| **209047_at** | *AQP1* | Hs.76152 | 358 | 7p14 | -1.23 |
| **215726_s_at** | *CYB5A* | Hs.465413 | 1528 | 18q23 | -1.23 |
| **203757_s_at** | *CEACAM6* | Hs.466814 | 4680 | 19q13.2 | -1.23 |
| **213317_at** | *CLIC5* | Hs.485489 | 53405 | 6p21.1-p12.1 | -1.24 |
| **203824_at** | *TSPAN8* | Hs.170563 | 7103 | 12q14.1-q21.1 | -1.25 |
| **201427_s_at** | *SEPP1* | Hs.275775 | 6414 | 5q31 | -1.26 |
| **210762_s_at** | *DLC1* | Hs.134296 | 10395 | 8p22 | -1.26 |
| **212741_at** | *MAOA* | Hs.183109 | 4128 | Xp11.3 | -1.29 |
| **206100_at** | *CPM* | Hs.654387 | 1368 | 12q14.3 | -1.29 |
| **212599_at** | *AUTS2* | Hs.654801 | 26053 | 7q11.22 | -1.31 |
| **219250_s_at** | *FLRT3* | Hs.41296 | 23767 | 20p11 | -1.32 |
| **209267_s_at** | *SLC39A8* | Hs.288034 | 64116 | 4q22-q24 | -1.34 |
| **208791_at** | *CLU* | Hs.436657 | 1191 | 8p21-p12 | -1.35 |
| **203021_at** | *SLPI* | Hs.517070 | 6590 | 20q12 | -1.35 |
| **201540_at** | *FHL1* | Hs.435369 | 2273 | Xq26 | -1.35 |
| **202295_s_at** | *CTSH* | Hs.148641 | 1512 | 15q24-q25 | -1.36 |
| **202149_at** | *NEDD9* | Hs.37982 | 4739 | 6p25-p24 | -1.36 |
| **210272_at** | *CYP2B7P1* | Hs.529117 | 1556 | 19q13.2 | -1.37 |
| **202768_at** | *FOSB* | Hs.590958 | 2354 | 19q13.32 | -1.37 |
| **208792_s_at** | *CLU* | Hs.436657 | 1191 | 8p21-p12 | -1.37 |
| **204424_s_at** | *LMO3* | Hs.504908 | 55885 | 12p12.3 | -1.40 |
| **39248_at** | *AQP3* | Hs.234642 | 360 | 9p13 | -1.40 |
| **37512_at** | *HSD17B6* | Hs.524513 | 8630 | 12q13 | -1.41 |
| **212951_at** | *GPR116* | Hs.362806 | 221395 | 6p12.3 | -1.41 |
| **205569_at** | *LAMP3* | Hs.518448 | 27074 | 3q26.3-q27 | -1.43 |
| **202992_at** | *C7* | Hs.78065 | 730 | 5p13 | -1.47 |
| **208250_s_at** | *DMBT1* | Hs.279611 | 1755 | 10q26.13 | -1.48 |
| **211689_s_at** | *TMPRSS2* | Hs.439309 | 7113 | 21q22.3 | -1.48 |
| **205776_at** | *FMO5* | Hs.642706 | 2330 | 1q21.1 | -1.49 |
| **203799_at** | *CD302* | Hs.130014 | 9936 | 2q24.2 | -1.51 |
| **201785_at** | *RNASE1* | Hs.78224 | 6035 | 14q11.2 | -1.51 |
| **205700_at** | *HSD17B6* | Hs.524513 | 8630 | 12q13 | -1.52 |
| **211597_s_at** | *HOPX* | Hs.654864 | 84525 | 4q11-q12 | -1.53 |
| **37004_at** | *SFTPB* | Hs.512690 | 6439 | 2p12-p11.2 | -1.61 |
| **209810_at** | *SFTPB* | Hs.512690 | 6439 | 2p12-p11.2 | -1.61 |
| **204811_s_at** | *CACNA2D2* | Hs.476273 | 9254 | 3p21.3 | -1.62 |
| **215454_x_at** | *SFTPC* | Hs.1074 | 6440 | 8p21 | -1.63 |
| **213936_x_at** | *SFTPB* | Hs.512690 | 6439 | 2p12-p11.2 | -1.66 |
| **214354_x_at** | *SFTPB* | Hs.512690 | 6439 | 2p12-p11.2 | -1.67 |
| **219747_at** | *C4orf31* | Hs.709520 | 79625 | 4q27 | -1.67 |
| **204712_at** | *WIF1* | Hs.284122 | 11197 | 12q14.3 | -1.69 |
| **208498_s_at** |  |  |  |  | -1.72 |
| **203913_s_at** | *HPGD* | Hs.596913 | 3248 | 4q34-q35 | -1.73 |
| **214135_at** | *CLDN18* | Hs.655324 | 51208 | 3q22.3 | -1.76 |
| **212950_at** | *GPR116* | Hs.362806 | 221395 | 6p12.3 | -1.80 |
| **205654_at** | *C4BPA* | Hs.1012 | 722 | 1q32 | -1.85 |
| **214433_s_at** | *SELENBP1* | Hs.632460 | 8991 | 1q21-q22 | -1.87 |
| **211548_s_at** | *HPGD* | Hs.596913 | 3248 | 4q34-q35 | -1.94 |
| **203914_x_at** | *HPGD* | Hs.596913 | 3248 | 4q34-q35 | -1.94 |
| **205261_at** | *PGC* | Hs.1867 | 5225 | 6p21.3-p21.1 | -1.96 |
| **218835_at** | *SFTPA2* | Hs.523084 | 729238 | 10q22.3 | -1.99 |
| **206754_s_at** |  |  |  |  | -2.05 |
| **219476_at** | *C1orf116* | Hs.32417 | 79098 | 1q32.1-q32.2 | -2.09 |
| **205725_at** | *SCGB1A1* | Hs.523732 | 7356 | 11q12.3-q13.1 | -2.13 |
| **204437_s_at** | *FOLR1* | Hs.73769 | 2348 | 11q13.3-q14.1 | -2.14 |
| **214199_at** | *SFTPD* | Hs.253495 | 6441 | 10q22.2-q23.1 | -2.16 |
| **209613_s_at** | *ADH1B* | Hs.4 | 125 | 4q21-q23 | -2.22 |
| **209612_s_at** | *ADH1B* | Hs.4 | 125 | 4q21-q23 | -2.37 |
| **38691_s_at** | *SFTPC* | Hs.1074 | 6440 | 8p21 | -2.42 |
| **214387_x_at** | *SFTPC* | Hs.1074 | 6440 | 8p21 | -2.51 |
| **211735_x_at** | *SFTPC* | Hs.1074 | 6440 | 8p21 | -2.58 |
| **205982_x_at** | *SFTPC* | Hs.1074 | 6440 | 8p21 | -2.62 |
| **210096_at** | *CYP4B1* | Hs.436317 | 1580 | 1p34-p12 | -2.92 |
