# Supplementary material for: Development and Validation of a Prognostic Gene-Expression Signature for Lung Adenocarcinoma
Source: PLoS One. 2012 Sep 7;7(9):e44225. doi: 10.1371/journal.pone.0044225 (PMC3436895; doi:10.1371/journal.pone.0044225)
Supplement: Table S2 — Drop in Concordance-index Score of Clinical Variables in ACC Cohort. (DOCX) [file pone.0044225.s007.docx]

**Table S2.** Drop in Concordance-index Score of Clinical Variables in ACC Cohort

| **Clinical Variables** | **Drop in C-index** | **95% CI** | ***p*-value** |
| --- | --- | --- | --- |
| Sex | 0.008 | -0.012 – 0.041 | 0.26 |
| Age | 0.005 | 0.01 – 0.033 | 0.34 |
| EGFR | 0.004 | -0.013 – 0.028 | 0.34 |
| KRAS | 0.003 | -0.01 – 0.025 | 0.35 |
| TP53 | 0.004 | -0.01 – 0.028 | 0.35 |
| Smoking | 0.002 | 0.011 – 0.021 | 0.41 |
| AJCC stage | 0.034 | -0.004 – 0.09 | 0.053 |
| Gene Signature | 0.042 | -0.007 – 0.1 | 0.029 |
